# C21orf91 Regulates Oligodendroglial Precursor Cell Fate—A Switch in the Glial Lineage?

**DOI:** 10.3389/fncel.2021.653075

**Published:** 2021-03-16

**Authors:** Laura Reiche, Peter Göttle, Lydie Lane, Paula Duek, Mina Park, Kasum Azim, Jana Schütte, Anastasia Manousi, Jessica Schira-Heinen, Patrick Küry

**Affiliations:** ^1^Department of Neurology, Medical Faculty, Heinrich-Heine-University Düsseldorf, Düsseldorf, Germany; ^2^CALIPHO Group, SIB Swiss Institute of Bioinformatics, Geneva, Switzerland; ^3^Department of Microbiology and Molecular Medicine, Faculty of Medicine, University of Geneva, Geneva, Switzerland

**Keywords:** white matter deficits, gliogenesis, cell fate, down syndrome, neuroregeneration

## Abstract

Neuropathological diseases of the central nervous system (CNS) are frequently associated with impaired differentiation of the oligodendroglial cell lineage and subsequent alterations in white matter structure and dynamics. Down syndrome (DS), or trisomy 21, is the most common genetic cause for cognitive impairments and intellectual disability (ID) and is associated with a reduction in the number of neurons and oligodendrocytes, as well as with hypomyelination and astrogliosis. Recent studies mainly focused on neuronal development in DS and underestimated the role of glial cells as pathogenic players. This also relates to C21ORF91, a protein considered a key modulator of aberrant CNS development in DS. We investigated the role of C21orf91 ortholog in terms of oligodendrogenesis and myelination using database information as well as through cultured primary oligodendroglial precursor cells (OPCs). Upon modulation of *C21orf91* gene expression, we found this factor to be important for accurate oligodendroglial differentiation, influencing their capacity to mature and to myelinate axons. Interestingly, C21orf91 overexpression initiates a cell population coexpressing astroglial- and oligodendroglial markers indicating that elevated C21orf91 expression levels induce a gliogenic shift towards the astrocytic lineage reflecting non-equilibrated glial cell populations in DS brains.

## Introduction

White matter, which makes up approximately 40% of the human brain, consists of axons, astrocytes, and myelin, the latter of which is imperative for stabilization, protection, and electrical insulation of axons enabling accelerated electrical signal propagation. In the adult central nervous system (CNS), myelin is generated by oligodendrocytes, either deriving from oligodendroglial precursor cells (OPCs) or niche-located adult neural stem cells (aNSCs; Akkermann et al., 2017). The structural integrity of myelin is of crucial importance for CNS function and restoration (Snaidero and Simons, 2014), making it vulnerable to pathological degeneration and inflammation (Waxman, 1992) or genetic intervention (Nave, 1994). Myelin loss, therefore, leads to impaired neuronal signaling, functional deficits, and shortened lifetime (Wilkins et al., 2003). Moreover, axonal nutrition has recently been shown to depend on myelin and oligodendrocytes (Simons and Nave, 2015). Hence, white matter deficits and myelin dysfunctions are considered a main contributing factor for neurodegenerative diseases and malfunctions of the CNS (Bercury and Macklin, 2015).

Down syndrome (DS) results from a trisomy of human chromosome (HSA) 21 and represents the most common genetic cause for cognitive impairments and intellectual disability (ID). The neurological profile of DS patients is characterized by hypotrophy and hypocellularity of neurons (reviewed by Stagni et al., 2017; Baburamani et al., 2019) and oligodendrocytes (Karlsen and Pakkenberg, 2011) accompanied by hypomyelination in the hippocampal formation (Abraham et al., 2012), whereas astroglial cell numbers are increased (Mito and Becker, 1993; Zdaniuk et al., 2011). DS astrocytes also show alterations in the structure and intracellular protein expression (Dossi et al., 2018). Moreover, structural and functional abnormalities of DS white matter were described (Fenoll et al., 2017).

Several studies indicate that white matter malformation contributes to neurological impairment in DS patients (Powell et al., 2014; Fenoll et al., 2017) and that hypomyelination is caused by a cell-autonomous phenomenon in oligodendrocyte development (Olmos-Serrano et al., 2016)—a research focus that has, nevertheless, not been paid much attention so far (Reiche et al., 2019). Olmos-Serrano et al. (2016) revealed a module (termed as M43) enriched in genes associated with oligodendrocyte differentiation and myelination to exhibit a distinct downregulation in several DS brain regions such as the hippocampus throughout development. Among genes such as *CNP*, *PLP1*, *SOX10*, and *GPR17*, also *C21ORF91* is listed within this cluster. Also known as *early undifferentiated retina and lens* (*EURL*), *C21ORF91* is localized at the centromeric boundary of the DS critical region (DSCR; encompassing 21q21-21q22.3) and was initially described to play a role in defective DS neurogenesis (Li et al., 2016). There, it was shown that *C21ORF91*’s transcript levels within the tested DS brain regions exhibited a spatiotemporal increase compared to equivalent controls. Furthermore, in DS lymphoblastoid cells, *C21ORF91* is overexpressed with a mean ratio close to 1.5, which is proportional to the gene dosage effect of trisomy 21, thus implied to be involved in the DS phenotype (Ait Yahya-Graison et al., 2007). Indeed, *C21ORF91* has previously been suggested to be relevant for the observed neurodevelopmental disorder and ID arising from HSA21 triplication (Slavotinek et al., 2000; Rost et al., 2004; Korbel et al., 2009; Li et al., 2016). Although expressed ubiquitously in healthy human tissues, C21orf91 protein was shown to be enriched in oligodendrocytes along with other proteins important for their differentiation such as Olig2 (Cahoy et al., 2008). Moreover, *C21ORF91* expression in the healthy human brain peaks between birth and adulthood (Li et al., 2016), hence coinciding with the onset and progression of myelination. Interestingly, cognitive deficits and hypomyelinated patterns in DS are thought to arise during these early stages of life and increase during development (Pennington et al., 2003; Rowe et al., 2006; Lanfranchi et al., 2010; Abraham et al., 2012). Based on these parallels as well as on the here presented functional data, a possible correlation between elevated C21orf91 expression levels and myelination deficits as observed in DS can be suggested.

## Materials and Methods

### Data Mining/Human C21ORF91 Correlations

RNA sequencing (RNAseq) and microarray data for humans were retrieved from Genevestigator (version 7.4.0; Hruz et al., 2008), a curated database that performs meta-analyses of gene expression data on a large panel of tissues and cell types of different experiments. For RNAseq data, 285 human tissues and cells were analyzed for their *C21ORF91* expression level. Gene expression was measured in transcripts per million (TMP). High expression as considered by Genevestigator corresponds to the log(2) of the average of the mean value higher than 3. For microarray data, 416 human tissues and cells were analyzed for their *C21ORF91* expression level. Gene expression is expressed as relative mean values. High expression as considered by Genevestigator corresponds to higher than 12.

*C21ORF91* RNAseq expression data on the human developmental and adult brain for 26 different brain structures expressed as the mean of reads per kilobase million (RPKM) values was retrieved from the Allen Brain Atlas (October 2019 and November 2020). Brain structures were grouped in *allocortex* (hippocampus); *basal ganglia* (striatum); *neural plate* (dorsolateral prefrontal cortex, ventrolateral prefrontal cortex, anterior (rostral) cingulate (medial prefrontal) cortex, orbital frontal cortex, primary motor-sensory cortex, parietal neocortex, posterior (caudal) superior temporal cortex (area 22c), inferolateral temporal cortex (area TEv, area 20), occipital neocortex, amygdaloid complex, upper (rostral) rhombic lip, temporal neocortex, the primary motor cortex (area M1, area 4), the primary somatosensory cortex (area S1, areas 3, 1, 2), posteroventral (inferior) parietal cortex, primary auditory cortex (core), primary visual cortex (striate cortex, area V1/17), cerebellum, cerebellar cortex) and *thalamus* (dorsal thalamus, mediodorsal nucleus of the thalamus). These grouped brain structures were analyzed for the following grouped ages: 8 and 9 post-conception weeks (pcw), 12 and 13 pcw, 16 and 17 pcw, 19 and 21 pcw, 24–26 pcw (24, 25 and 26 pcw), 35–37 pcw, 4 and 10 months, 1–4 (1, 2, 3 and 4 years), 8–15 (8, 11, 13 and 15 years), 18–23 (18, 19, 21 and 23 years) and 30–40 (30, 36, 37 and 40 years). Data from the *ventricular zone* (lateral ganglionic eminence, medial ganglionic eminence, caudal ganglionic eminence) was available only for 8 and 9 pcw and was therefore omitted.

To obtain additional data on *C21ORF91* expression in the nervous system, we used the Brain EXPression Database (BrainEXP; Jiao et al., 2019), which performs a meta-analysis of microarray and RNAseq data in a subset of nervous system tissues consisting of 4567 samples from 2863 healthy individuals gathered from public databases and their data. The spinal cord, substantia nigra, hippocampus, hypothalamus, amygdala, putamen, caudate nucleus, nucleus accumbens, frontal cortex, BA9, anterior cingulate cortex, cerebellum, and cerebellar hemisphere could be analyzed and compared.

The heatmap displayed in the Allen Brain Atlas database (Sunkin et al., 2013) representing z-scores values of the microarray data for six adult donors was surveyed to identify structures with the highest *C21ORF91* expression. The four probes refer to *C21ORF91* sequences on the microarray chip: A_23_P211015 (GGT GAG GTA GAG CAA CTG AAT GCA AAG CTC CTA CAG CAA ATC CAG GAA GTT TTT GAA GAG); A_24_P125839 (AGT AGG GCG AAC AGG AAT GAA GTC GCA CCT ACC CAT AAA CAA CTG ACC TAA ACA GAC TT), CUST_5965_PI416261804 (ATT CGA TGA CTC TTG GTG AGG TAG AGC AAC TGA ATG CAA AGC TCC TAC AGC AAA TCC AG) and CUST_5966_PI416261804 (GAA AAA AGA AGA GAC AAT CTC TAG TCC AGA GGC TAA TGT CCA GAC CCA GCA TCC ACA TTA).

To characterize *C21ORF91* expression on a cellular level, single-nucleus RNAseq data (Allen Brain Atlas) was collected from two sets. The first covers multiple adult human cortical areas (MCA) including the middle temporal gyrus, anterior cingulate cortex, primary visual cortex, primary motor cortex, primary somatosensory cortex, and primary auditory cortex. The second experiment included specifically the adult primary motor cortex (M1). Single-nucleus RNAseq is expressed as counts per million (cpm) trimmed means.

To set-up a coexpressed gene cluster for *C21ORF91*, a list of genes whose expression positively correlates with *C21ORF91* in the adult brain was extracted using the “Correlate Gene Search” functionality appended to the microarray expression data from adult donors provided by the Allen Brain Atlas. Also, coexpression data from the BrainEXP database (Jiao et al., 2019) was retrieved with the default parameters. The genes coexpressing with *C21ORF91* were further analyzed to identify if they coexpress with each other.

Gene ontology (GO) enrichment analysis on the coexpression set from the adult brain microarray from Allen Brain Atlas was performed using PANTHER [(Thomas et al., 2003, 2006); RRID: SCR_004869; released 20190711] including GO biological process, molecular function and cellular component terms.

*C21ORF91* homologs were searched by protein BLAST at NCBI. Reciprocal best hits were considered as orthologs. Homology in jawless fishes was searched by tBLASTn with human and zebrafish protein sequences as queries against RefSeq Genome and nucleotide collection databases.

### Gene Expression Heatmap Generation

Bulk transcriptomic datasets assembled as done previously were used for defining the expression of *D16Ertd472* mouse *C21orf91* ortholog, further referred to as *C21orf91*) across multiple cell types that are present in the forebrain (Azim et al., 2017, 2018), and Gene Expression Omnibus (GEO) repository IDs are stated. These included substages of postnatal oligodendroglia (GSE9566; P16); early postnatal neural stem cells (NSCs) and transiently amplifying progenitors (TAPs; GSE60905); young adult substages of NSCs (GSE54653); young adult neuroblasts and ependymal cells (GSE18765); young adult choroid plexus cells (GSE82308); young adult and postnatal astrocytes (GSE35338, GSE9566); young adult microglia (GSE58483) and embryonic day 14 radial glial cells (vRGS; GSE40582). All analyses were performed in RStudio using publicly available packages installed directly from the Bioconductor consortium^1^. The LIMMA package was used to incorporate all datasets described above systematically which were then subsequently normalized using the standard RMA method. Known oligodendroglial, astrocyte, and NSC lineage markers were additionally studied for heatmap plotting purposes by taking the most significant probes for each gene (differential expression by <0.0001 False Discovery Rate between the individual cell types studied). The selected genes were visualized in an unsupervised heatmap using the pHeatmap package^2^.

### Brain Tissue Preparation, Sectioning, and Immunohistochemistry

For the analysis of transcript and protein levels of *RGD1563888* (rat *C21orf91* ortholog, further referred to as *C21orf91*), embryonic day 16 (E16), postnatal day 0 (P0), P7, P25, and adult (2–3 months) Wistar rats of either sex were deeply anesthetized and killed by an overdose of isoflurane (Piramal-Healthcare, Mumbai, India). Rat brains were isolated, shortly surface-washed with ice-cold Dulbecco’s phosphate-buffered saline (PBS; Sigma-Aldrich, St. Louis, USA), and then frozen in −35°C to −50°C methyl butane and stored at −80°C until further processing. Regions of interest including whole hemisphere (HS), forebrain (FB), corpus callosum (CC), hippocampal formation (HF), and cerebellum (CB) were sectioned coronally using a cryostat (Leica CM3050S) at −28°C. Out of 50–100 μm thick slices, smaller regions (CC and HF) were isolated using a pre-cooled scalpel within the cryostat chamber. Note, that P0 tissue sections were used exclusively for Western blot analysis. Tissues were stored at −80°C.

For immunohistochemistry, P7 brains were directly processed, while adult rats were transcardially perfused with 150 ml ice-cold Dulbecco’s phosphate-buffered saline (PBS; Sigma-Aldrich, St. Louis, MO, USA) followed by 400 ml 4% paraformaldehyde (PFA). Rat brains were harvested and post-fixed for 2 days (P7 brains) or overnight (adult brains) in 4% PFA at 4°C, followed by 48 h cryoprotective dehydration in 30% sucrose (in PBS) at 4°C. Brains were embedded in TissueTek OCT (Sakura Finetek Europe, Netherlands), frozen in −35°C to −50°C methyl butane, and stored at −80°C until preparation of 14 μm coronal sections using a cryostat (Leica CM3050S). Sections were stored at −80°C.

Immunohistochemical staining was performed as previously described (Beyer et al., 2020). Briefly, thawed brain sections were air-dried for at least 15 min at room temperature (RT), rehydrated in distilled water for 5 min, transferred to −20°C acetone for 5 min, and washed in 1× Tris-buffered saline (TBS; pH 7.6) and 1× TBS-T (TBS containing 0.02% Triton X-100) for 5 min each. Non-specific staining was blocked with 10% biotin-free bovine serum albumin (BSA; in TBS-T) for 1 h at RT, followed by application of the following antibodies (in 10% BSA in TBS) and incubation overnight at 4°C: rabbit anti-C21orf91 [1:1,000, Santa Cruz Biotechnology, Cat# sc-83610 (Li et al., 2016)], rabbit anti-C21orf91 (1:200, Bioss, Cat# bs-9983R), rabbit anti-C21orf91 (1:300, Sigma-Aldrich, Cat# HPA049030), mouse anti-neuronal nuclei antigen (NeuN; 1:1,000, Merck Millipore, Cat# MAB377), goat anti-PDGFR (alpha/CD140A, 1:250, Neuromics, Cat# GT15150), mouse anti-oligodendrocyte transcription factor 2 (Olig2; 1:500, Merck Millipore, Cat# MABN50), guinea pig anti-glial fibrillary acidic protein (GFAP; 1:2,000, Synaptic Systems, Cat# 173004), and mouse anti-adenomatous polyposis coli for oligodendrocytes (APC, CC1; 1:500, GeneTex, Cat# GTX16794). Sections were washed two times for 5 min in TBS and incubated with the species-appropriate fluorochrome-conjugated secondary antibodies (1:200 in PBS; donkey: anti-goat; goat: anti-rabbit, anti-mouse, anti-guinea pig; Alexa Fluor488- or Alexa Fluor594-conjugated) and DAPI (4,6-diamidino-2-phenylindole; nuclei labeling, 0.04 μl/ml; Roche Diagnostic GmbH) for 1 h at RT. Slices were mounted with Immu-Mount (Thermo Fisher Scientific, Darmstadt, Germany) and analyzed using a confocal laser scanning microscope 510 (CLSM 510, Zeiss, Jena, Germany) and the ImageJ BioVoxxel software (Schindelin et al., 2012). Z-stacked tile scans on average eight brain slices per marker and time-point (two slices per animal) were fused to a maximum intensity projection *via* ImageJ. C21orf91-positive cells within the whole tile scan projection were counted and normalized to its area [mm^2^], distinguishing between CC and surrounding gray matter structures (GM). Then, on the one hand, the average distribution of C21orf91 expressing cells within the population of marker-positive cells (such as of all GFAP expressing cells within a tile scan) was calculated. To depict the marker-specific population size/density, pie charts vary in size. On the other hand, the mean percentage of marker expressing cells within the C21orf91-positive population was evaluated per animal. Rat brain preparations were approved by the ZETT (Zentrale Einrichtung für Tierforschung und Wissenschaftliche Tierschutzaufgaben; O69/11, V54/09).

### Rat Oligodendroglial Cell Culture

Based on the procedure of McCarthy and de Vellis (1980), the generation of primary OPC cultures from postnatal day zero to one (P0–1) cerebral rat cortices of Wistar rats (either sex) was performed as previously described (Kremer et al., 2009; Göttle et al., 2018) whereas the cell culture medium was supplemented with fetal bovine serum (FBS) from a different company (Capricorn Scientific, Palo Alto, CA, USA). Primary OPCs (>97% pure) were either seeded onto 0.25 mg/ml poly-D-lysine coated (PDL, Sigma-Aldrich) glass coverslips (13 mm) in 24-well plates (for immunocytochemistry; 2.5 × 10^4^ cells/well) or 0.25 mg/ml PDL coated 24-well plates (for quantitative reverse transcription-polymerase chain reaction (qRT-PCR); 5 × 10^4^ cells/well) in high-glucose DMEM-based Sato medium. After 1.5 h, cell differentiation was induced by changing medium to differentiation medium (Sato medium supplemented with 0.5% FBS). The medium was exchanged every 3 days. The preparation of rodent primary oligodendroglial cell cultures was approved by the ZETT (O69/11, V54/09).

### Plasmid Construction and OPC Transfection

Plasmid design and generation were conducted by Hybrigenics SA, Paris, France and kindly provided to our lab. Briefly, the complete coding sequence of the rat ortholog *C21orf91* (*RGD1563888*) was inserted into the company’s pV22 vector, which is an equivalent vector to pHTN (Promega France; eucaryotic expression vector containing a HaloTag sequence) with slightly different polylinkers. OPCs were transfected *via* electroporation using the basic glia nucleofector kit and a nucleofector II device (both Lonza, Basel, Switzerland). In detail, 0.8–1 × 10^6^ cells were transfected using the high-efficiency program A-033 resuspended in 100 μl nucleofection solution and a total amount of 2 μg plasmid per 1 × 10^6^ cells. For visualization of short-term experiments, control (pHTN) and C21orf91 overexpression vectors were co-transfected with pmaxGFP (Lonza; a green fluorescent protein expression vector) and for visualization of transplanted cells, vectors were co-transfected with the pcDNA3-hyg-citrine vector (yellow fluorescent protein; Kremer et al., 2009) in a ratio of 10:1. Transfected OPCs were seeded onto 0.25 mg/ml PDL coated glass coverslips (13 mm) in 24-well plates (for immunocytochemistry; 7 × 10^4^ cells/well) or 0.25 mg/ml PDL coated 24-well plates (for qRT-PCR; 1.5 × 10^5^ cells/well) in expansion medium (Sato medium supplemented with 10 ng/ml recombinant human basic fibroblast growth factor (bFGF) and 10 ng/ml recombinant human platelet-derived growth factor-AA (PDGF-AA; both R&D Systems, Wiesbaden-Nordenstadt, Germany). After 4–5 h, the medium was exchanged to a differentiation medium (Kremer et al., 2009).

### RNA Preparation, cDNA Synthesis, and Quantitative RT-PCR

Total RNA purification from tissues was done using Trizol reagent (Invitrogen) while cultured cells were lysed using 350 μl RLT lysis buffer (Qiagen) supplemented with β-mercaptoethanol (1:100, Sigma) and total RNA was purified by using RNeasy Mini Kit (Qiagen, Hilden, Germany) according to manufacturer instructions including DNase digestion. Before quantitative real-time polymerase chain reaction (qPCR), reverse transcription with 250 ng RNA [measured using a NanoDrop ND 1000 (Peqlab, Erlangen, Germany)] was done using the High-Capacity cDNA Reverse Transcription Kit (ThermoFisher Scientific, Darmstadt, Germany). Gene expression levels were determined on a 7900HT sequence detection system (Applied Biosystems) applying SybrGreen universal master mix (ThermoFisher Scientific, Darmstadt, Germany). For sequence detection, the following forward (fwd) and reverse (rev) primers, generated *via* PrimerExpress 2.0 software (Applied Biosystems), were used, with β-actin (ACTB) and glyceraldehyde 3-phosphate dehydrogenase (GAPDH) serving as reference genes: ACTB_fwd: AACCCTAAGGCCAACCGTGAAA, ACTB_rev: AGTGGTACGACCAGAGGCAT, C21orf91_fwd: CTTCAGCAAGCGTCATCGAATT, C21orf91_rev: GTATCCTGGAAGACGCGGATG, CNPase_fwd: ATGCTGAGCTTGGCGAAGAA, CNPase_rev: GTACCCCGTGAAGATGGCC, GAPDH_fwd: GAACGGGAAGCTCACTGGC, GAPDH_rev: GCATGTCAGATCCACAACGG, MBP_fwd: CAATGGACCCGACAGGAAAC, MBP_rev: TGGCATCTCCAGCGTGTTC, MOG_fwd: CAGTTGTCACGCAGCTACGC, MOG_rev: ATGCCCTGGCCCTATCACTC. Relative gene expression levels were determined according to the ΔΔCt method (ThermoFisher Scientific, Darmstadt, Germany). All measurements were done in duplicates; generated from *n* = 8 independent experiments and data are shown as mean values ± SEM.

### Immunocytochemistry and Assessment of Morphology

To evaluate marker expression and morphological maturation, the immunocytochemical analysis was performed after cells were fixed using 4% paraformaldehyde (PFA) at RT for 10 min. Non-specific binding of antibodies was prevented by incubation in blocking solution [10% normal goat serum (NGS); in PBS containing 0.1% Triton X-100] at RT for 45 min. Subsequently, cells were subjected to primary antibody solution (10% NGS, in PBS containing 0.01% Triton X-100), using the following dilutions overnight at 4°C: rabbit anti-GFAP (1:1,000, DAKO Agilent, Cat# Z0334), mouse anti-GFAP (1:1,000, Merck Millipore, Cat# MAB3402), mouse anti-2′, 3′-cyclic-nucleotide 3′-phosphodiesterase (CNPase; 1:1,000, Biolegend, Cat# 836402), rat anti-myelin basic protein (MBP; 1:250, Bio-Rad Laboratories, Cat# MCA409S), rabbit anti-C21orf91 (1:200, Bioss, Cat# bs-9983R), rabbit anti-C21orf91 (1:300, Sigma-Aldrich, Cat# HPA049030), mouse anti-CC1 (1:1,000, GeneTex, Cat# GTX16794), mouse anti-Olig2 (1:500, Merck Millipore Cat# MABN50), rabbit anti-Olig2 (1:500, Merck Millipore, Cat# AB9610), mouse anti-myelin oligodendrocyte glycoprotein (MOG; 1:500, Merck Millipore, Cat# MAB5680), goat anti-PDGFR (alpha/CD140A, 1:250, Neuromics, Cat# GT15150), mouse anti-astrocyte cell surface antigen-1 (ACSA-1/GLAST; 1:200, Miltenyi, Ca# 130-095-822), rabbit anti-hairy and enhancer of split-1 (HES1; 1:250, Invitrogen, Cat# PA5-28802), mouse anti-NK2 Homeobox 2 (NKX2.2; 1:100, R&D Systems, Cat# 883411), rabbit anti-sex-determining region Y-box 10 (Sox10; 1:100, S1058C002, DCS Immunoline, RRID: AB_2313583), chicken anti-green fluorescent protein/citrine (GFP; 1:1,000; Aves Labs, Cat# GFP-1020). Following three washing steps with PBS, secondary antibodies (anti-mouse, anti-rabbit, anti-goat) conjugated with either Alexa Fluor405, Alexa Fluor488, or Alexa Fluor594 (1:500; Thermo Fisher Scientific, Darmstadt, Germany) in PBS supplemented with DAPI (0.02 μg/ml; Roche Diagnostic GmbH, Mannheim, Germany) were applied for 90 min at RT. Cells were mounted with Citifluor (Cilifluor, Leicester, United Kingdom). For image acquisition, the Zeiss Axionplan2 microscope (Zeiss, Jena, Germany) was used and the analysis was performed with the ImageJ BioVoxxel software (Schindelin et al., 2012). Nine images per coverslip (2 coverslips/condition; mean of 2 coverslips generated from the same animal pool represents *n* = 1 independent experiment) were captured using 20× magnification and the same exposure times throughout each experiment and marker expression strength study. For quantification, the number of marker-positive cells in relation to DAPI-positive- (total number, for non-transfected cells) or GFP expressing cells (for transfected cells) was calculated and shown as a percentage. To assess the degree of morphological maturation, transfected (green fluorescent) OPCs were analyzed by fluorescence microscopy (Zeiss Axioplan, Jena, Germany) as previously described (Kremer et al., 2009; Göttle et al., 2010). Based on morphological cell parameters (processes, branches), cells were distinguished into 3 different categories for morphological maturation starting with a low number of processes in progenitor cells to multiple process-bearing cells (low, medium) up to more mature cells with a high degree of arborization or even sheath building (high). For classification of hybrid cells—cells with oligodendroglial- and astroglial marker coexpression—ubiquitous [indicating astrogenesis (Setoguchi and Kondo, 2004)] as well as nuclear Olig2-, Sox10-, Nkx2.2- and strong CC1 expression was correlated with GFAP- and GLAST expression.

### Western Blotting

Isolated CC and HF tissues were lysed using an Ultra-turrax disperser (IKA^®^-Werke GmbH and Co. KG, Staufen, Germany) and radioimmunoprecipitation assay buffer (RIPA buffer, Cell Signaling Technology, Danvers, MA, USA) supplemented with HALT^TM^ Protease-/Phosphatase inhibitor cocktail and EDTA (both Thermo Fisher Scientific). Afterward, sonication with an ultrasound homogenizer (SonopulsHD2070, 50% power, pulse 0.5 s on and 0.5 s off) was performed for 10 s and samples were centrifuged (14,000 rpm, 10 min, 4°C) to proceed with supernatants. Protein concentrations were determined using the DC Protein Assay (BioRad). Specimens were subjected to standard sodium dodecyl sulfate (SDS) gel electrophoresis and semi-dry western blotting using Bolt 12% Bis-Tris Plus gels and nitrocellulose membranes (both Thermo Fisher Scientific). Blocking was confirmed by total protein staining using the Pierce^TM^ Reversible Protein Stain Kit (Thermo Fisher Scientific) also used for protein normalization. Afterward, membranes were blocked with Superblock (in TBS, Thermo Fisher Scientific) for 1 h at RT and applying the following primary antibodies: rabbit anti-C21orf91 [1:1,000, Santa Cruz Biotechnology, Cat# sc-83610 (Li et al., 2016)], rabbit anti-C21orf91 (1:300, Sigma-Aldrich, Cat# HPA049030), mouse anti-GAPDH (1:5,000, Merck Millipore Cat# MAB374) and the secondary antibodies anti-rabbit IgG, HRP-linked (1:2,000, Cell Signaling Technology Cat# 7074,) and anti-mouse IgG (H + L), made in horse (1:5,000, Vector Laboratories, Burlingame, CA, USA Cat# PI-2000). For visualization, Super Signal West Pico Chemiluminescent Substrate (Thermo Fisher Scientific) was applied for 5 min. To ensure reliable quantification, membranes were stripped with 10 ml ReBlot Plus Strong Solution (1×, Merck Millipore) to detect C21orf91 and the housekeeping protein (GAPDH) sequentially on the same membrane. Protein bands were quantified using the Fusion FX software (Vilber Lourmat, Eberhardzell, Germany). The intensity for each band was determined and normalized to the total amount of the loaded protein amount and the intensity of the GAPDH band of the corresponding sample. Quantification was repeated two times for the published C21orf91 antibody (Li et al., 2016) and two times for the C21orf91 antibody from Sigma-Aldrich. Both antibodies marked a protein band of the same size and therefore the mean across all Western blot experiments (*n* = 3 animals) was calculated.

### Myelinating Co-cultures, OPC Transplantation, and Assessment of Myelination Capacity

Dissociated neuron/oligodendrocyte co-cultures were obtained from E16 rat cerebral cortices (Wistar rats of either sex) as previously described in Göttle et al. (2015) and Göttle et al. (2018) with the only difference that 9 × 10^4^ cortical cells were plated per well. After 15 days *in vitro* (DIV15), 10 × 10^4^ transfected OPCs per coverslip were plated directly in the center of a co-culture. The medium was exchanged twice a week with freshly prepared myelination medium until DIV25. Then, co-cultures were fixed with 4% paraformaldehyde for 15 min at RT and processed for immunofluorescent staining. Blocking solution contained 2% NGS and 0.5% Triton X-100 in PBS, whereas the following primary antibodies were diluted in 2% NGS and 0.1% Triton X-100: mouse anti-MBP (1:250, Biolegend, San Diego, CA, USA, Cat# 836504), mouse anti-CC1 (1:800, GeneTex, Cat# GTX16794), rabbit anti-GFAP (1:800, DAKO Agilent, Cat# Z0334). After washing with PBS, secondary antibodies (anti-mouse and anti-rabbit) conjugated with either Alexa Fluor405 or Alexa Fluor594 (1:500; Thermo Fisher Scientific, Darmstadt, Germany) in PBS supplemented with DAPI (0.02 μg/ml; Roche Diagnostic GmbH, Mannheim, Germany) were applied for 90 min at RT. To assess the degree of cellular maturation, only transfected (green fluorescent) cells were scored by applying an evaluation tool based on morphological cell parameters (processes, branches). Moreover, expression and distribution of the MBP protein were assessed and led to the categorizations: MBP-positive cells with a high degree of arborization [pos], integrated and myelinating oligodendrocytes displaying T-shape structures (myelin; Göttle et al., 2015), non-organized MBP expressing [NOM] cells, characterized by a rather disorganized MBP accumulation and unusual morphologies as well as MBP-negative cells [neg]. For quantification, the number of protein marker-positive cells in relation to GFP expressing cells (transfected cells) was calculated and shown as percentage (*n* = 9 experiments). The generation of rodent myelinating co-cultures was approved by the LANUV (Landesamt für Natur, Umwelt und Verbraucherschutz; Az.81-02.04.2018.A388).

### Statistical Analysis

Data are presented as mean values ± standard error of the mean (SEM). Graphs and statistical analysis were performed using Excel and the GraphPad Prism 8.0.2 software (GraphPad Prism, San Diego, CA, USA; RRID: SCR_002798). Shapiro-Wilk normality test was used to assess the absence of Gaussian distribution of all datasets. To determine statistical significance for normally distributed data sets, the Students *t*-test was applied for comparing two groups and one-way analysis of variance (ANOVA) with Turkey post-test for multiple comparisons was applied to compare three or more groups. For data sets not passing the Shapiro–Wilk normality test, Mann–Whitney U test for comparing two groups, and Kruskal–Wallis test with Dunn’s post-test for multiple comparisons of three or more groups was applied. Statistical significance thresholds were set as follows: *p ≤ 0.05; **p ≤ 0.01; ***p ≤ 0.001 and ns = not significant. “*n*” represents the number of independent experiments.

## Results

C21orf91 was previously shown to affect neurogenesis during fetal brain development and suggested to impact neuropathogenesis of HSA21-related disorders such as DS (Li et al., 2016). Interestingly, this study also revealed the highest *C21ORF91* mRNA expression levels in the adult corpus callosum (CC) which is considered as the largest white matter structure in the brain. This prompted us to investigate C21orf91’s correlations and expression patterns in available databases and to study functional consequences upon forced overexpression in the oligodendroglial lineage.

### The Human C21ORF91 Expression Profile Strongly Correlates With the Oligodendroglial Lineage

**Figure 1:**
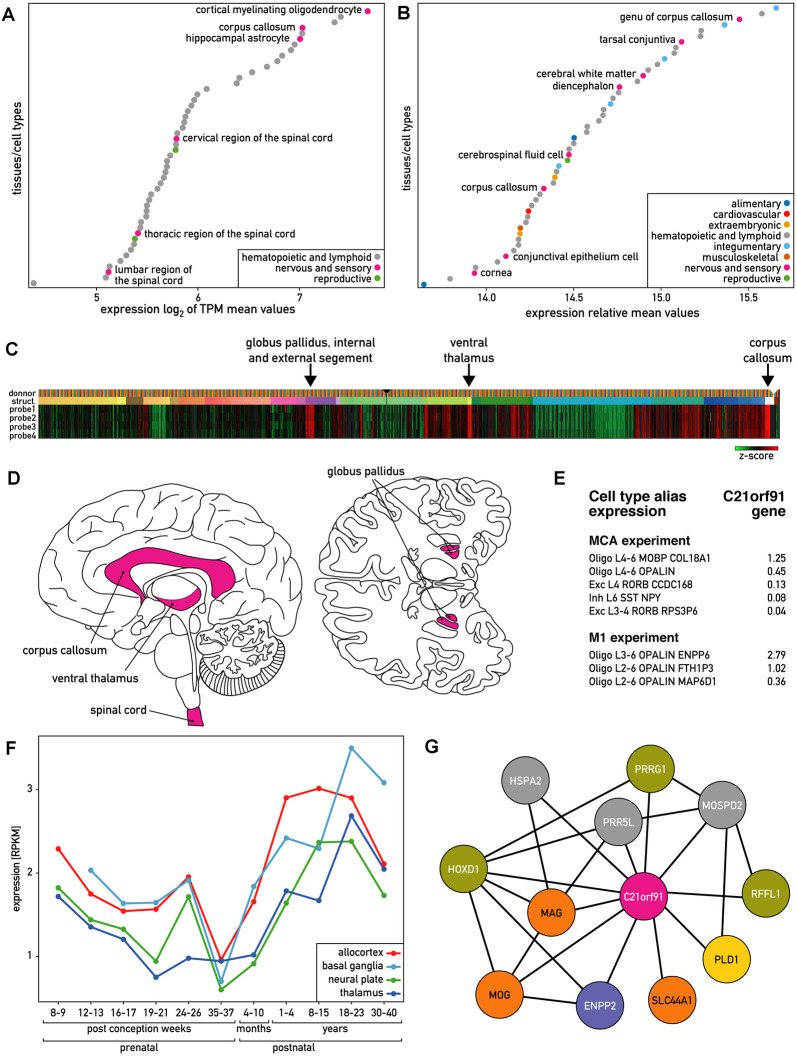
Expression of human *C21ORF91* gene. **(A)** The 50 tissues and cell types with the highest *C21ORF91* expression according to Genevestigator measured with RNAseq and expressed as the log(2) of TPM (transcripts per kilobase million) mean values. Cortical myelinating oligodendrocytes and hippocampal astrocytes are the cell types, and corpus callosum and spinal cord are the tissues that are most enriched for *C21orf91*. **(B)** The 50 tissues and cell types with the highest C21ORF91 expression according to Genevestigator measured with microarrays and expressed as relative mean expression values. Corpus callosum, diencephalon, and cerebral white matter are the nervous system structures most enriched for *C21orf91*. **(C)** Heat map visualization of microarray expression data from six adult donors for the four available probes for* C21ORF91* from the Allen Brain Atlas. Indicated with arrows are the structures with the highest expression. Data is displayed as z-scores. Probe 1–4 correspond to *C21ORF91* sequence A_23_P211015, A_24_P125839, CUST_5965_PI416261804 and CUST_5966_PI416261804. **(D)** Schematic sagittal and inferior view of the human brain indicating the regions where *C21ORF91* gene is expressed according to Genevestigator and Allen Brain Atlas. **(E)** Single-nucleus RNAseq data from Allen Brain Atlas expressed as count per million (cpm) trimmed means. MCA stands for multiple cortex area set of experiments and M1 for primary motor cortex experiment. L4–6, L6, L3–4, L2–6, L3–6, L4 refer to cortex areas. Oligo stands for oligodendrocytes, Exc for excitatory neuron, and Inh for inhibitory neuron. **(F)** *C21ORF91* expression profile depicts a wave-like pattern, progressively increasing after birth to the age of 23 years. The expression is monitored for different brain structures at different ages. RNAseq data expressed as the mean of RPKM values. Ages vary from prenatal [8–37 post-conception weeks (pcw)] to postnatal stages from 4–10 months and 1–40 years. Brain structures were grouped as follows: *allocortex* (red) = hippocampus; *basal ganglia* (light blue) = striatum; *neural plate* (green) = 19 structures including frontal cortex and cerebellum; *thalamus* (dark blue) = dorsal thalamus, mediodorsal nucleus of thalamus. Data from the *ventricular zone* are not included. **(G)** Coexpression network of genes correlating with *C21ORF91* expression and each other generated from BrainEXP database. Nodes are colored according to protein function: orange: myelination, purple: oligodendrogenesis, yellow: dendrite spine morphogenesis, green: other expression evidence in the nervous system, and gray: other functions.

According to Genevestigator (Hruz et al., 2008), human *C21ORF91* is ubiquitously and highly expressed. Out of 285 human tissues and cells with available RNA sequencing (RNAseq) data, 167 tissues (60%) demonstrated high expression levels (log_(2)_ > 3 of an average of the mean value of TPM). Based on these RNAseq data, hematopoietic cells and the nervous system including corpus callosum and different regions of the spinal cord belong to the 50 tissues and cells showing the highest expression of *C21ORF91* (Figure 1A). Microarray data from this platform, which encompasses a wider panel of brain structures than the RNAseq data, further indicated that within the nervous system; corpus callosum, diencephalon, and cerebral white matter belong to the most *C21ORF91* enriched regions (Figure 1B). In agreement with these observations, microarray expression data from 6 human adult donors from the Allen Brain Atlas (Sunkin et al., 2013) confirmed the highest expression levels in the corpus callosum and ventral thalamus and globus pallidus, both part of the diencephalon (Figure 1C). To obtain additional data on *C21ORF91* expression in the nervous system, we used the Brain EXPression Database (BrainEXP; Jiao et al., 2019). According to this database, *C21ORF91* expression is highest in the spinal cord, a structure that is absent in the panel analyzed by the Allen Brain atlas. The nervous system regions expressing *C21ORF91* (pink) are summarized in Figure 1D.

At the cellular level, RNAseq data from Genevestigator showed that *C21ORF91* is enriched in cortical myelinating oligodendrocytes and hippocampal astrocytes (Figure 1A). Single nucleus RNAseq data retrieved from the Allen Brain Atlas show that *C21ORF91* is preferentially expressed in oligodendroglial (Oligo) as compared to neuronal populations (Exc, Inh; Figure 1E). The oligodendroglial cell population Oligo derived from cortex areas L3–6 (Oligo L3–6 OPALIN ENPP6) which exhibits the highest *C21ORF91* expression also features both, high *OPALIN* transcript levels, encoding a protein involved in oligodendrocyte terminal differentiation (de Faria et al., 2019), as well as *ENPP6* transcripts, encoding a marker of newly forming oligodendrocytes (Xiao et al., 2016). RNAseq expression data from human brain structures at different ages indicate that *C21ORF91* expression is initiated at eight postconceptional weeks (pcw), then decreases until birth and increases progressively until the age of 23 years before it declines again (Figure 1F), thus approximately resembling the wave-like myelination process during brain development. Interestingly, the phylogenetic distribution of *C21ORF91*, shown to be conserved in various Gnathostomata (jawed vertebrates)—including mammals, chicken, Xenopus, or Zebrafish, but absent in jawless fish (Supplementary Figure 1)—closely mirrors the one of myelin (Baumann and Pham-Dinh, 2001).

Both, BrainEXP and the Allen Brain Atlas provide coexpression data. BrainEXP indicates that *C21ORF91* coexpresses with eleven genes (Figure 1G). The list includes *MOG*, *SLC44A1* and *MAG*, *HOXD1*, and *ENPP2/autotaxin* of which all were shown to be involved in oligodendrogenesis and myelination (Booth et al., 2007; Wheeler et al., 2015). It also includes *PRRG1* which is uncharacterized but is among the top 50 genes overexpressed in non-activated adult OPCs compared with activated adult OPCs (Moyon et al., 2015). The other coexpressed genes are *PLD1*, which promotes dendritic spine morphogenesis *via* *PKD1* activation (Li et al., 2019), *RFFL* which is reported to be expressed in the corpus callosum, brain stem, spinal cord and cerebellar white matter, and *HSPA2* and *PRR5L*, for which we did not find evidence for function in nervous system processes, oligodendrogenesis or myelination.

Additionally, the “Correlate Gene Search” of the Allen Brain Atlas was applied to adult brain microarray data indicating that C21ORF91 is coexpressed with 51 proteins, including ENPP2, HSPA2, MOG, PLD1, PRR5L, PRRG1, and SLC44A1 (with a Pearson’s correlation >= 0.85). The analysis of GO terms associated with these 51 proteins revealed an enrichment of the following terms: myelination (GO:0042552), ensheathment of neurons (GO:0007272), axon ensheathment (GO:0008366), glial cell differentiation (GO:0010001), gliogenesis (GO:0042063), and myelin sheath (GO:0043209).

### C21orf91 Ortholog Expression Is Enriched in White Matter Regions and Maturing Oligodendrocytes During Rodent Brain Development

**Figure 2:**
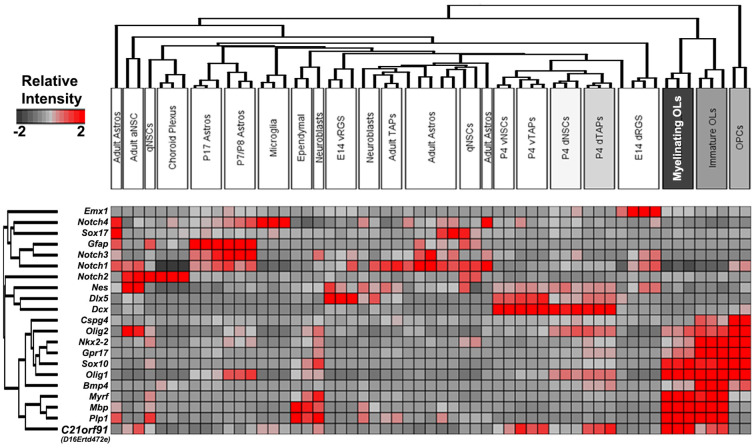
Validation of enriched expression of mouse *C21orf91* ortholog in later stage oligodendroglial lineage cells compared to multiple cell types present in the forebrain. Bulk transcriptomic datasets assembled with essential hallmark constructed as a heatmap with known cell-specific hallmark genes and *C21orf91* ortholog (*D16Ertd472e*) clusters readily with later stage oligodendroglial markers and is highly expressed in myelinating oligodendrocytes (OL) as well as in transiently amplifying progenitors (TAPs) derived from region-specific early postnatal subventricular zone (SVZ) microdomains. Note: some datasets [e.g., adult astrocytes (Astros)] are not clustered together based on the genes lists for unsupervised clustering. The color legend represents the relative expression strength with red color indicating high values and gray color displaying lower values. Abbreviations: aNSC, activated neural stem cell; qNSC, quiescent NSC; Astros, astrocytes; vRGS/NSCs/TAPs, ventral radial glial cells/NSCs/TAPs; dNSCs/TAPs/RGS, dorsal NSCs/TAPs; OLs, oligodendrocytes; and OPCs, oligodendroglial precursor cells.

**Figure 3:**
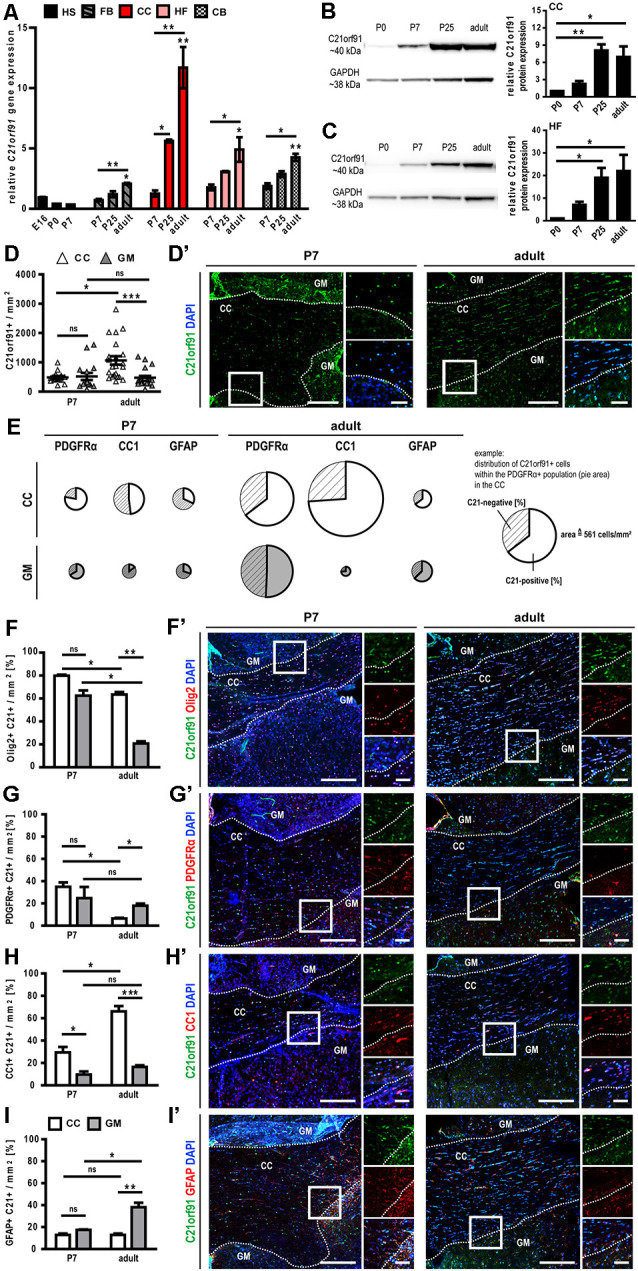
C21orf91 ortholog expression is enriched in white matter structures and correlates with OPC differentiation markers during rat brain development. **(A)** Time- and region-specific qRT-PCR revealed enriched *C21orf91* ortholog (*RGD1563888*) transcript levels in the corpus callosum (CC, red bars) and hippocampal formation (HF, rose bars) compared to hemisphere (HS, black bars), forebrain (FB, gray dashed bars) and cerebellum (CB, chessboard bars), thereby showing an increasing pattern from embryonic day 16 (E16) over postnatal days (P7, P25) up to adult. Note, that stars without significance bars (adult bars) indicate a significant increase from E16 to adult. For a clear presentation, the indication of additional significance bars has been omitted. A similar correlation was depicted for C21orf91 ortholog protein abundance using Western blotting for CC **(B)** and HF **(C)**. Results derived from *n* = 3 animals **(A–C)** and measurements were repeated twice per lysate **(B,C)**. Note, that P0 tissue sections were used entirely for Western blot analysis. P7 and adult rat brain sections were immunostained for C21orf91 (RGD1563888; **D′**), and oligodendroglial markers **(F′–H′)**, and an astroglial marker **(I′)**. The number of C21orf91+ cells **(D)** was enriched in the adult corpus callosum (CC; white triangles) compared to the surrounding gray matter (GM; gray triangles) and CC of P7 rats. Panel **(D’)** shows representative tile scans for the analyzed region in P7 and adult rat brain sections stained for C21orf91 (green) with nuclei in blue (DAPI). The average distribution of C21orf91-positive cells within the PDGFRα-, CC1- or GFAP-positive cell population is shown in **(E)**. The marker-related population size/density is depicted by the size (=area) of the pie charts. White pies represent cells of the CC and gray pies refer to GM located cells. The whole circle area of the white PDGFRα pie chart (see example) equals 561 PDGFRα-positive cells/mm^2^ and represents the scale for the other pie charts. C21orf91 expression is highly correlated with Olig2 **(F)**, PDGFRα **(G)** and CC1 **(H)** expression in the CC and GFAP **(I)** expression in adult GM. Representative images of tile scans and blow-ups are shown in **(F′)** for Olig2 (red), **(G′)** for PDGFRα (red), **(H′)** for CC1 (red) and **(I′)** for GFAP (red). Nuclei are shown in blue (DAPI). White dashed lines depict the border of CC and surrounding GM. Squares refer to blow-ups. Scale bars for tile scans: 200 μm, scale bars for blow-ups: 50 μm. Data are shown as mean values (±SEM) deriving from *n* = 4 animals; Kruskal-Wallis test with Dunn’s post-test: *p ≤ 0.05, **p ≤ 0.01, ***p ≤ 0.001, ns = not significant.

To corroborate human findings, rodent *C21orf91* ortholog expression (*D16Ertd472e* for mouse, *RGD1563888* for rat) was examined using recently generated bulk transcriptomic datasets of numerous purified cell types and workflows for their analysis (Azim et al., 2015, 2018; Figure 2). This analysis confirmed the anticipated enrichment of *C21orf91*’s expression in myelinating oligodendrocytes. Note that *C21orf91* subclustered most with known mature oligodendrocyte markers, *MBP, Plp1*, and *Myrf*, and forming a larger cluster comprising pan oligodendrocyte or earlier stage lineage markers which include *Olig2, Sox10, Nkx2.2*, and *Gpr17*. As for a functional assessment we intended to use rat primary OPCs as previously established and published (Kremer et al., 2009; Göttle et al., 2010, 2015, 2018), we additionally examined *C21orf91* expression during rat CNS development. To this end, brains of postnatal day 0 (P0), P7, P25, and 2–3 months old (adult) rats were prepared and transcript and protein expression levels were analyzed in a region-specific way using tissues from the forebrain (FB), corpus callosum (CC), hippocampal formation (HF) and cerebellum (CB; Figures 3A–C). Real-time quantitative RT-PCR determination demonstrated that *C21orf91* gene expression was highest in adult CC compared to the other regions and revealed a clear transcript increase over time in all tested brain regions, again most apparent in CC (Figure 3A). This could be further verified *via* Western blot analysis, showing that C21orf91 protein expression was enriched during development in CC and HF (Figures 3B,C), peaking at P25 (observed with both tested antibodies, and shown as mean value).

As a next step, immunohistochemical staining of coronal P7 and adult rat CC brain sections was conducted. We assessed the antibody used by Li et al. (2016) as well as several other commercially available antibodies in our immunocytochemistry and confirmed that C21orf91 expression is correlating with neuronal marker NeuN expression in cortical sections for all of them (data not shown). Subsequent staining experiments were then nevertheless conducted using the published antibody (Li et al., 2016), to ensure comparability. Evaluation of staining patterns revealed that the density of C21orf91 expressing cells per mm^2^ was enriched in adult CC compared to the surrounding gray matter (GM; Figures 3D,D’). In the next step, the distribution of C21orf91-positive cells within lineage-specific marker populations was assessed (Figure 3E). PDGFRα hereby represents the OPC population, CC1 accounts for mature oligodendrocytes, and GFAP was used as an astroglial marker. Interestingly, except for small populations in the early developmental stage P7 (see pie area; CC1-positive cells in the GM (gray pie) and GFAP-positive cells in CC (white pie) and GM), between 48–80% of oligodendroglial or astroglial lineage cells expressed C21orf91 in P7 and adult CC and GM. In a further survey, the distribution of nuclear Olig2- (Figure 3F), PDGFRα- (Figure 3G), CC1- (Figure 3H), and GFAP-positive cells (Figure 3I) within the C21orf91 expressing cell populations were analyzed. In CC, 80% of the C21orf91 expressing cells in P7 and 60% of the C21orf91 expressing cells in adult rats were also Olig2-positive (Figures 3F,F’). Furthermore, almost 40% of the C21orf91 expressing cells in P7 CC were PDGFRα-positive, declining in the adult CC along the course of white matter development (Figures 3G,G’). In parallel, the degree of CC1/C21orf91 expressing cells increased over time (Figures 3H,H’), additionally depicted by a proportional increase of C21orf91-positive cells within the CC1 population (compare CC1 in P7 and adult; Figure 3E). Interestingly, the percentage of C21orf91 expressing astrocytes also increased in the surrounding GM structures during development, comprising up to 40% of the C21orf91-positive cells (Figures 3I,I’). Furthermore, the proportional distribution of C21orf91 expressing cells within the GFAP-positive population doubled during development in both, CC and GM (Figure 3E).

### C21orf91 Ortholog Expression Correlates With Differentiation and Maturation of Cultured OPCs

**Figure 4:**
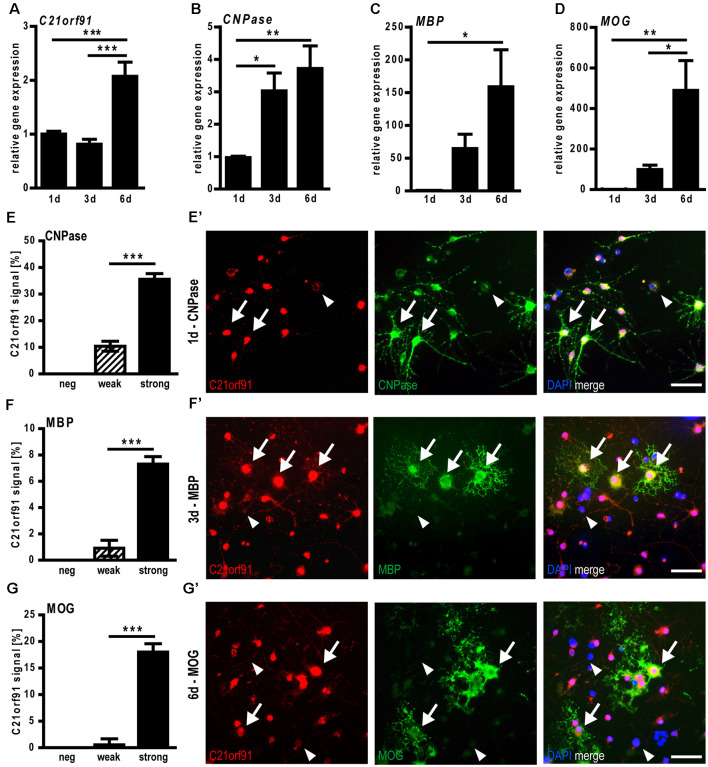
C21orf91 ortholog expression correlates with myelin expression during spontaneous differentiation of cultured rat primary oligodendroglial precursor cells (OPCs). Determination of transcript levels using qRT-PCR indicated an upregulation of *C21orf91*ortholog (*RGD1563888*; **A**) concurrent with increased expression of *CNPase* **(B)**, *MBP* **(C)**, and *MOG* **(D)** for 6 days (1d-6d). Stage-specific analysis of C21orf91 ortholog (RGD1563888) protein expression (red, Bioss anti-C21orf91 antibody) *via* double immunostaining performed for CNPase at 1d **(E,E′)**, MBP at 3d **(F,F′)** and MOG at 6d **(G,G′)** revealed that maturation/myelin marker expression during differentiation highly correlates with strong cellular C21orf91 expression levels (black bars). Arrows point to cells with a strong C21orf91 expression that are also myelin-positive, arrowheads point at weak/no expressors that are myelin-negative. Blue nuclei represent DAPI staining. Note, that there are no myelin-positive cells that lack C21orf91 expression. Scale bars: 50 μm. Data are shown as mean values (±SEM) deriving from *n* = 8 experiments **(A–D)** and *n* = 5 experiments **(E–G)**. Statistical significance was calculated using one-way ANOVA with Tukey post-test **(A–D)** and Mann-Whitney *U* two-tailed test **(E–G)**: *p ≤ 0.05, **p ≤ 0.01, ***p ≤ 0.001.

To investigate the role of C21orf91 in oligodendroglial differentiation, we first examined the expression of differentiation-associated markers and C21orf91 during spontaneous differentiation of cultured primary rat OPCs. Transcript levels of *C21orf91* (Figure 4A) were mildly downregulated at day 3, where *CNPase* expression (Figure 4B) is already significantly upregulated, but then upregulated at day 6, similar to the induction of *MBP* and* MOG* expression (Figures 4C,D). As oligodendroglial cell differentiation is also reflected by the induction of specific myelin protein expression at particular time points (1d, 3d, 6d), double immunofluorescent staining with antibodies directed against C21orf91, CNPase (Figures 4E,E’), MBP (Figures 4F,F’) and MOG (Figures 4G,G’) were conducted. Discriminating between different C21orf91 expression strengths (strong: black bars, weak: dashed bars and cells without any positivity), it could be demonstrated that most oligodendroglial cells were expressing C21orf91, with the majority of them featuring strong C21orf91 expression levels (see representative images in Figures 4E’,F’,G’). Note that especially cells with strong C21orf91 signals (Figures 4E–G) also displayed expression of the stage-specific markers and that none of the myelin-positive cells was negative for C21orf91.

### C21orf91 OIrtholog Overexpression Influences Rat OPC Differentiation

**Figure 5:**
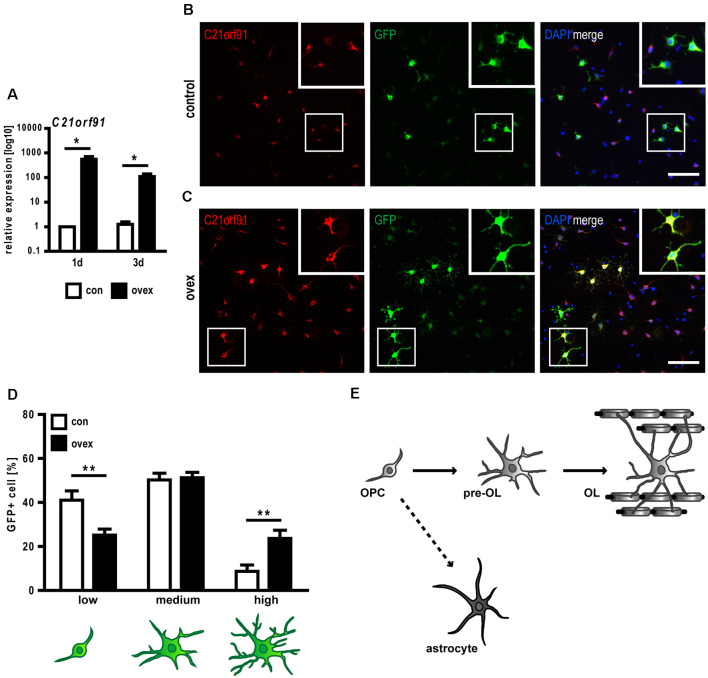
C21orf91 ortholog overexpression accelerates morphological maturation of OPCs. OPCs were co-transfected with a GFP expression (for visualization, green) and an empty control vector (con, white bars) or a C21orf91 ortholog (RGD1563888) overexpression construct (ovex, black bars). Overexpression was confirmed using qRT-PCR **(A)** and immunostaining for C21orf91 (red, anti-C21orf91 antibody from Sigma-Aldrich) protein after 2 days of differentiation **(B,C)**. Squares show the blow-ups of representative transfected cells which indicate that overexpressing cells in **(C)** exhibited enriched C21orf91 protein abundance. Additionally, their distinct morphological maturation is highlighted (compare cell morphologies in **B,C**) which was further assessed using process- and branch-dependent categories (low-high), indeed revealing an accelerated morphological maturation **(D)**. Cell nuclei were labeled by DAPI (blue). Scale bars: 100 μm. Data are shown as mean values (±SEM) deriving from *n* = 3 experiments **(A)** and *n* = 8 experiments **(D)**. Statistical significance was calculated using Mann-Whitney *U* two-tailed test **(A)** and Student’s two-sided, unpaired *t*-test **(B)**: *p ≤ 0.05, **p ≤ 0.01. OPCs, despite their primarily designated development into myelinating oligodendrocytes (OL), can also differentiate into astrocytes **(E)**.

Given that the occurrence and strength of differentiation markers correlated with strong C21orf91 ortholog expression levels, it was of interest to find out whether C21orf91 ortholog modulation influences oligodendroglial differentiation, particularly in the context of increased C21orf91 levels in DS. To this end, C21orf91 ortholog (RGD1563888) was overexpressed leading to elevated transcript and protein levels (Figures 5A–C). Of note, transfection experiments were conducted with C21orf91 overexpression constructs (black bars) and the corresponding empty control vector (white bars) along with a green fluorescent protein (GFP) expression vector for detection of modulated cells. Morphological assessment of transfected cells was carried out according to our previously published schemes (Kremer et al., 2009; Göttle et al., 2010, 2015) and demonstrated that overexpression of C21orf91 accelerated the maturation process (in terms of process growth and arborization) resulting in a significantly increased number of cells with more mature morphologies (Figures 5B–D).

Although OPCs are generally determined to give rise to oligodendrocytes, they also exert a certain potential to generate astrocytes both *in vitro* (Rao and Mayer-Proschel, 1997; Nishiyama et al., 2009) as well as *in vivo* (Aguirre and Gallo, 2004; Guo et al., 2009; Tanner et al., 2011; see Figure 5E). Interestingly, an increased astrocyte generation (Mito and Becker, 1993; Zdaniuk et al., 2011) along with a diminished myelin formation is observed in DS (Abraham et al., 2012). We, therefore, investigated whether C21orf91 overexpression impacts the lineage fate by depicting the expression changes of several astroglial and oligodendroglial differentiation markers (Table 1). This analysis revealed that C21orf91 overexpression resulted in a distinct reduction of cells exhibiting oligodendroglial features such as nuclear Olig2- and strong Nkx2.2 expression. On the other hand, overexpressing cells increased the expression of PDGFRα compared to control transfected cells. However, CC1 expression appeared to be far appeared to be far less abundant (40% reduction) in modulated cells again. Additionally, Sox10 nuclear localization (Rehberg et al., 2002) appeared to be affected by C21orf91 overexpression as the number of cells with exclusively nuclear signals was decreased. On the other hand, an overall induction of astroglial markers was observed. Cytoplasmic translocation of Olig2 is known to account for astroglial differentiation (Setoguchi and Kondo, 2004), and indeed cell numbers with ubiquitous (nuclear and cytoplasmic) Olig2 expression were strongly increased throughout differentiation upon C21orf91 overexpression. Similarly, Hes1-positivity, a transcription factor known to induce astrogliogenesis at the expanse of oligodendrogenesis (Wu et al., 2003), was also elevated in cells with forced C21orf91 expression. Moreover, an overall upregulation of GFAP-positive cells at days 2, 3, and 5 of differentiation was observed leading to a 33%-increase in the number of GLAST expressing cells at day 5 (Table 1).

**Table 1:** C21orf91 ortholog (RGD1563888) overexpression results in a dysbalanced expression profile of glial differentiation markers during OPC differentiation.

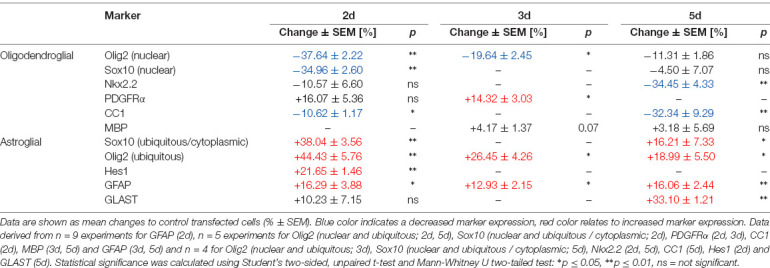

### Overexpression of C21orf91 Ortholog Results in Aberrant Coexpression of Oligodendroglial and Astroglial Differentiation Markers

**Figure 6:**
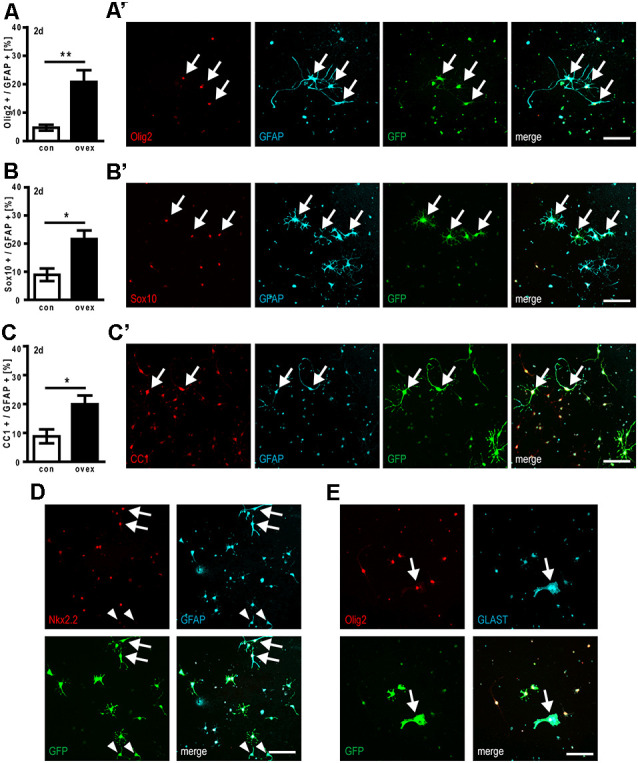
C21orf91 ortholog overexpression results in aberrant OPC differentiation creating hybrid cells coexpressing astroglial and oligodendroglial markers. OPCs were co-transfected with a GFP expression (for visualization, green) and an empty control vector (con, white bars) or a C21orf91 ortholog (RGD1563888) overexpression construct (ovex, black bars). After 2 days (2d) of differentiation and C21orf91 overexpression, a significantly increased number of cells expressing GFAP (cyan) with oligodendroglial markers (red) Olig2 **(A)**, Sox10 **(B)**, and CC1 **(C)** were observed, representative images of which are shown in **(A’–C’)**. Additional aberrant coexpression was also observed for GFAP (cyan) and Nkx2.2 (red; **D**) and for GLAST (cyan) with Olig2 (red; **E**). Arrows point at double-positive hybrid cells, arrowheads in **(D)** point to “normal” cells exhibiting a GFAP-positivity in the expected absence of oligodendroglial markers such as Nkx2.2. Scale bars: 100 μm. Data are shown as mean values (±SEM) deriving from *n* = 5–7 experiments. Mann-Whitney U two-tailed test: *p ≤ 0.05, **p ≤ 0.01.

We next studied to what degree cellular coexpression of astroglial- and oligodendroglial markers could be observed. Immunocytofluorescent staining demonstrated that the forced expression of C21orf91 ortholog initiated aberrant combinations such as (nuclear) Olig2, Sox10, and CC1 expression in combination with GFAP signals at day 2 of differentiation (Figures 6A’–C’). Compared to control transfected cells, the numbers of Olig2/GFAP expressing cells were more than tripled (Figure 6A), whereas Sox10/GFAP expressing (Figure 6B) and CC1/GFAP-positive cells (Figure 6C) were doubled upon overexpression. In C21orf91 overexpressing cells, this mixed phenotype could occasionally still be found after 5 days in culture (data not shown). Furthermore, also coexpression of Nkx2.2 together with GFAP, as well as a few cells displaying a ubiquitous expression of Olig2 [indicating astrogenesis (Setoguchi and Kondo, 2004)] together with GLAST were found (Figures 6D,E).

### Rat Oligodendroglial Cells Display Accelerated Maturation but Diminished Myelination Capacity Upon C21orf91 Ortholog Overexpression

**Figure 7:**
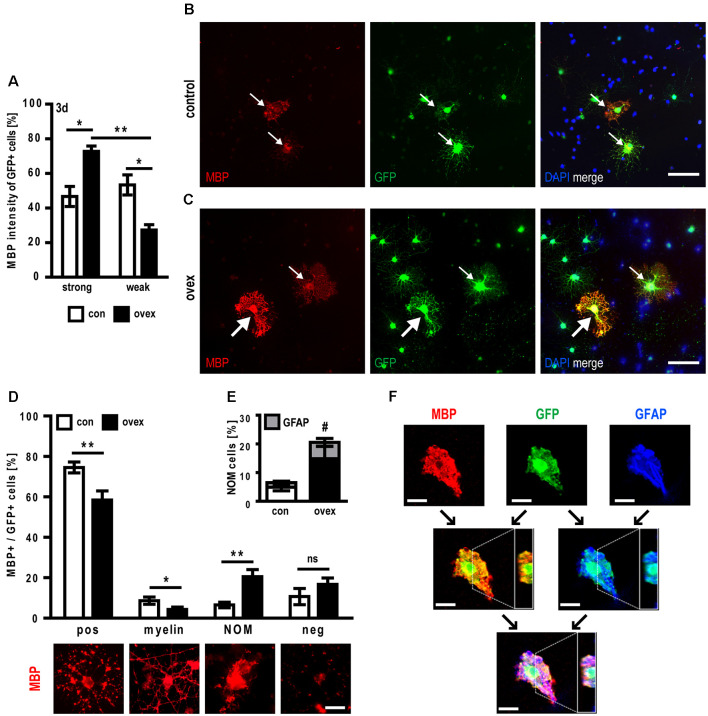
C21orf91 ortholog overexpression leads to myelination failures and abnormal GFAP coexpression in myelin-producing oligodendrocytes. Immunofluorescence analysis of co-transfected OPCs with a GFP expression vector (for visualization, green) and an empty control vector (con, white bars) or a C21orf91 ortholog (RGD1563888) overexpression construct (ovex, black bars) revealed that after 3 days (3d) of differentiation the proportion of cells with strong MBP expression was increased upon C21orf91 overexpression **(A)**. While control transfected cells exhibited MBP vesicle-like signals (red; **B**) C21orf91 overexpression resulted in stronger MBP signals (bold arrows) and myelin protein presence in cell processes **(C)**. Thin arrows point at cells with weak MBP expression. Cell nuclei were labeled by DAPI (blue). To assess whether the observed elevated levels of produced MBP protein correlate with the establishment of functional myelin, transfected OPCs were transplanted onto and cultured for 10 days on dissociated co-cultures. By categorizing GFP-positive cells (green) into different MBP expression- and morphological-related stages **(D)**, it was shown that C21orf91 overexpression resulted in fewer MBP expressing (pos) cells and also diminished their capacity to ensheath axons (myelin). They rather exhibited strong but non-organized MBP expression (NOM). Double immunostaining for MBP and GFAP (gray bars, blue stain) exposed NOM cells also to aberrantly coexpress both markers **(E)** which were confirmed *via* the orthogonal projection of a confocal, z-stacked microscopy image **(F)**. Black arrows show which channels were merged; white dashed lines depict the y-axis cut through the z-stack which is shown in the white box on the right side of the images. Scale bars: 50 μm **(B,C)**; 25 μm **(D)** and 20 μm **(F)**. Data are shown as mean values (±SEM) deriving from *n* = 5 **(A)** and *n* = 9 experiments **(D,E)**. Statistical significance was calculated using Kruskal-Wallis test with Dunn’s post-test **(A)** and Student’s two-sided, unpaired *t*-test **(D,E)**: *p ≤ 0.05, **p ≤ 0.01, ns = not significant; ^#^*p* ≤ 0.05 for the statistical analysis of GFAP + /MBP+ cells (gray bars) in **(E)**.

As C21orf91 ortholog overexpression leads to dysregulated OPC differentiation and the acquisition of non-permissive marker combinations, their maturation capacity was evaluated. We first observed, that upon C21orf91 overexpression, cells did not show a significant difference for the positivity of maturation marker MBP (Table 1). However, the expression strength of MBP was increased after 3 days of differentiation when compared to control cells (Figure 7A). Following the observed accelerated morphological maturation at day 2 of differentiation (Figure 5D), C21orf91 overexpressing cells exhibited morphologically elaborated maturation (Figure 7C) also at day 3 compared to control transfected cells (Figure 7B). Furthermore, control cells displayed vesicle-like MBP signals (Figure 7B), indicating MBP expression is still at an earlier stage as compared to C21orf91 overexpressed cells. Next, C21orf91 overexpressing OPCs were evaluated in a more physiological environment allowing axon/oligodendrocyte interactions to occur and we assessed whether the produced MBP protein could contribute to the generation of functional myelin. For this purpose, we used myelinating neuron-oligodendrocyte co-cultures (Göttle et al., 2015, 2018, 2019) onto which transfected primary rat OPCs were applied during the myelination process. Transplanted cells were maintained in co-culture for another 10 days in presence of a myelination-inducing medium and then assessed for their potential to integrate and to ensheath axons (detectable as T-shaped MBP-positive structures) as previously established for p57kip2 suppressed OPCs in our former studies (Göttle et al., 2015). We distinguished between MBP-negative cells (neg), MBP expressing oligodendrocytes (pos), oligodendrocytes myelinating axons (myelin, T-shapes), and non-organized MBP expressing (NOM) cells. NOM cells were characterized by a rather disorganized MBP accumulation and by a somehow collapsed morphology (Figures 7D,F). We found that OPCs with enforced C21orf91 expression failed to ensheath axons when compared to control transfected cells (Figure 7D). Also, the number of NOM cells was substantially increased (Figure 7D). Of note, GFAP/MBP double staining revealed that some of these NOM cells exhibited non-permissive coexpression of these two proteins (Figures 7E,F).

## Discussion

There is an increasing acceptance of the fact that white matter composition and functionality contribute to a healthy and functional CNS and that respective deficiencies are therefore likely implicated in many if not all neurological manifestations (Kremer et al., 2016). And indeed, an unusual, aberrant glial composition has been observed in the CNS of DS patients which has been suggested to contribute to developmental deficiencies and subsequent cognitive impairments and ID (Haydar and Reeves, 2012; Kanaumi et al., 2013; Stagni et al., 2017; Dossi et al., 2018). However, the underlying reasons why the glial compartment is affected by chromosome 21 triplication and which of the dysregulated genes contribute to glial malformation remain to be understood.

The *C21ORF91* gene has so far not been investigated in the context of oligodendrogenesis and white matter, although it was listed within the dysregulated gene network M43 in DS which is associated with oligodendroglial development and myelination (Olmos-Serrano et al., 2016). Our here presented studies demonstrate that *C21ORF91* correlates with white matter formation and oligodendroglial lineage. Not only being conserved in various jawed vertebrates (Supplementary Figure 1), closely mirroring the phylogenetic distribution of myelin (Baumann and Pham-Dinh, 2001), our bioinformatical analyses independently revealed a coexpression network of genes including *PLD1*, *MOG*, *MAG*, *OPALIN, SLC44A1* and *ENPP2/autotaxin* (Figure 1G), all of which are associated with oligodendrogenesis, myelination, glial cell differentiation and axon ensheathment, and also being part of Olmos-Serrano et al.’s (2016) M43 gene cluster. Furthermore, we here confirmed this bioinformatics-based correlation of human expression data for rodents, demonstrating on the one hand that C21orf91 ortholog was indeed enriched in the CC, the largest white matter structure of the CNS while revealing on the other hand, that it was also clearly associated with oligodendroglial differentiation and maturation. Moreover, here we show that slight disturbances in the expression strength as introduced by overexpression, reproducing conditions of the developing CNS in DS (Ait Yahya-Graison et al., 2007; Li et al., 2016) appear to interfere with OPC maturation and the proper establishment of myelin sheaths while creating cells with aberrant expression profiles. Because forced overexpression led to faster OPC maturation (seen by MBP expression strength; Figure 7), yet defective myelination, complex patterns of hypomyelination in DS could be explained, with myelin markers MBP and MOG depicting non-significant differences in the expression in early periods of life, then being progressively downregulated in DS patients (Abraham et al., 2012; Olmos-Serrano et al., 2016). Based on these observations, we suggest that overexpression of this gene contributes to hypomyelination as described in DS.

In this regard, it is tempting to speculate that the glial disbalance, more precisely the overpopulation of astroglial cells, such as described in the hippocampus of infant and adult DS brains (Mito and Becker, 1993), is related to enriched *C21ORF91* expression. We confirmed increased levels of C21orf91 ortholog expression in the hippocampal formation (HF) during rat brain development and showed that in addition to myelinating oligodendrocytes, *C21ORF91* is also substantially expressed in hippocampal astrocytes. Furthermore, elevated levels of C21orf91 ortholog augmented the expression of astroglial markers in differentiating OPCs and even resulted in cells displaying both lineage features. To what degree C21orf91-dependent aberrant astrogenesis from OPCs contributes to a DS-related overpopulation of astrocytes and whether also glial precursor cells committed to the astroglial lineage are additionally dysregulated, remains to be addressed in future studies.

Related to the here described significantly increased combinations of astroglial and oligodendroglial markers in OPCs with elevated *C21orf91* ortholog expression levels, it is reasonable to assume that such cells are conflicted in their differentiation paths. It remains therefore to be shown in future studies to what extent this is a transient phenomenon with cells exhibiting a possible extension of early glial progenitor virtues. Consequently, whether and when progression into either astroglial or delayed oligodendroglial lineages occurs—possibly inflicting successful distribution, tissue integration, and axon interactions—or whether such cells die or become actively omitted within the developing CNS needs also to be investigated. Moreover, to understand how such aberrant expression patterns arise, it will be necessary to investigate responsible signaling pathways, thus allowing the identification of altered dynamics in DS. As summarized recently (Reiche et al., 2019), several signaling pathways are potentially involved in neural/glial fate decision and differentiation in DS. Of note, in terms of neurogenesis, Li et al. (2016) already demonstrated neural β-catenin upregulation in response to overexpression of *C21orf91*. Noteworthy, the Wnt/β-catenin signaling is also a key regulator of oligodendrocyte development, as it is transiently activated in OPCs concurrent with the initiation of terminal differentiation (Emery, 2010). It will therefore be of interest to see whether C21orf91-dependent β-catenin regulation also accounts for glial cells and which other suspected molecules are responding to elevated C21orf91 levels in OPCs. In this regard, studies on axin2/catenin are worth mentioning since these molecules are already investigated in the context of white matter lesions in human newborns with neonatal ischemic and gliotic brain damage. Axin2 is a target of Wnt and negatively feeds back on this pathway, thereby promoting degradation of β-catenin (Fancy et al., 2011).

Further investigations along this line will be the subject of upcoming studies and could indeed contribute to the identification of therapeutic approaches which would allow to support or protect white matter development in young DS patients—a so far clinically unmet need. In this regard, it is worth mentioning that myelin repair studies in the context of multiple sclerosis (MS) or after hypoxia-ischemia mediated preterm brain injury have advanced in the last decade and that several modulating pharmacological agents are currently tested in clinical trials (Kremer et al., 2011, 2016; Reiche et al., 2019). Most promising substances emanating from demyelinating disease research should therefore be evaluated for their potential to correct the here described non-permissive cellular phenotype and to promote cells in their process to contact and myelinate axons.

Likewise, when it comes to the misexpression of MBP protein in *C21orf91* ortholog overexpressing cells in a more physiological context, it will also be of interest to study mechanisms of translational control in distal regions of oligodendrocyte processes. Regulated *via* ribonucleoprotein complexes referred to as RNA granules (Maggipinto et al., 2004) and with its mRNA residing in a translationally inactive state, the role of for example ribonucleoprotein types F and A2 (hnRNP_F, hnRNP_A2), both involved in post-transcriptional regulation of MBP expression (White et al., 2012) should be examined. Of note, fetal DS brains were found to possess increased protein levels of hnRNP_A2/B1 which was suggested to lead to impaired MBP expression (Kim et al., 2001). The here observed accumulation of MBP protein around oligodendroglial somata (NOM cells; Figures 7D,F) could therefore result from deficits in the transport of the mRNA-packed granules, suggesting that C21orf91 could be involved in specific transport processes. Such an extended functional role is supported by C21orf91’s association with microtubules^3^.

Finally, the here proposed influence of the C21orf91 protein on the establishment of mainly oligodendrocytes and white matter needs to be confirmed in suitable *in vivo* paradigms. Existing mouse models for DS such as the commonly used strains Ts65Dn and Ts1Cje comprise several HSA21 homologous genes located on mouse chromosome 16 (MMU16; Antonarakis, 2017; Herault et al., 2017) and are therefore likely not to support this strategy as they would not allow focusing exclusively on the functionality of the *C21ORF91* gene. Furthermore, Li et al. (2016) highlighted that this gene is also not represented within the modulated regions of those strains. Likewise, *C21orf91* ortholog knockout animals would also not be suitable, given that our data on oligodendroglia indicate that the observed cellular phenotype is a consequence of non-physiologically elevated gene expression levels. Such a constellation, therefore, needs to be mimicked by a *C21orf91* ortholog transgenic overexpression model, which needs yet to be generated. Here, an inducible expression might be considered to avoid too high protein levels as well as to provide the possibility to apply different time windows of gene induction. Nevertheless, a valuable alternative to such *in vivo* studies would be given by the generation of induced pluripotent stem cell (iPSC) generated neural cells that can be fostered to become oligodendrocytes [as shown by us in Jadasz et al. (2018)]. C21orf91 overexpression in such cells could be investigated in a tissue environment such as organoids and using DS patient-derived cells, it could then also be expanded towards humans. Of note, *C21ORF91* was classified as the second most induced gene among 71 genes overexpressed in DS iPSCs (Chou et al., 2012). If such increased levels are maintained in neural/oligodendroglial progeny cells molecular reverting/correcting strategies could be applied to assess the pathophysiological functionality of this gene.

## Data Availability Statement

The datasets presented in this study can be found in online repositories. The names of the repository/repositories and accession number(s) can be found in the article/Supplementary Material.

## Ethics Statement

The animal study was reviewed and approved by LANUV; Landesamt für Natur, Umwelt und Verbraucherschutz.

## Author Contributions

LR, PG, and PK contributed to the conception and design of the study. LL, PD, and KA analyzed and presented data from existing databases and websites. LR, MP, JS, and AM performed experiments. LR, MP, LL, PD, KA, JS-H, PG, AM, and PK contributed to data analysis and interpretation. LR, AM, and MP performed the statistical analysis. LR, LL, PD, KA, PG, and PK contributed to the data visualization. LL, PD, and KA contributed written sections of the manuscript. LR and PK wrote the manuscript. LR, PG, and PK contributed to funding acquisition. PK supervised the project. All authors contributed to the article and approved the submitted version.

## Conflict of Interest

The authors declare that the research was conducted in the absence of any commercial or financial relationships that could be construed as a potential conflict of interest.

## Footnotes

^1^https://www.bioconductor.org/packages/devel/workflows/vignettes/arrays/inst/doc/arrays.html

^2^https://cran.r-project.org/web/packages/pheatmap/index.html

^3^http://www.proteinatlas.org/ENSG00000154642-C21orf91/antibody

## Supplementary Material

SUPPLEMENTARY FIGURE 1 Multiple sequence alignment of human *C21ORF91* and model organism orthologs performed with ClustalO and Jalview.

## Abbreviations

ACSA-1/GLAST: astrocyte cell surface antigen-1
ACTB: ß-actin
aNSC(s): adult neural stem cell(s)
APC/CC1: adenomatous polyposis coli protein (antibody clone CC1)
BFGF: basic fibroblast growth factor
BSA: bovine serum albumin
CC: corpus callosum
CNS: central nervous system
CNPase: 2′,3′-cyclic-nucleotide 3′-phosphodiesterase
DIV: days *in vitro*
DS: Down syndrome
ENPP2/autotaxin: ectonucleotide pyrophosphatase/phosphodiesterase 2
EURL: early undifferentiated retina and lense
FB: forebrain
FBS: fetal bovine serum
GAPDH: glyceraldehyde 3-phosphate dehydrogenase
GEO: Gene Expression Omnibus
GFAP: glial fibrillary acid protein
GFP: green fluorescent protein
GM: gray matter
Gpr17: G protein-coupled receptor-17
Hes1: hairy and enhancer of split-1
HF: hippocampal formation
HOXD1: homeobox protein Hox-D1
HRP: horse radish peroxidase
HS: hemisphere
HSA21: human chromosome 21
HSPA2: heat shock protein family A (Hsp70) member 2
ID: intellectual disability
iPSC: induced pluripotent stem cell
MAG: myelin associated glycoprotein
MBP: myelin basic protein
MMU16: mouse chromosome 16
MOG: myelin oligodendrocyte glycoprotein
MS: multiple sclerosis
Myrf: myelin regulatory factor
NeuN: neuronal nuclei antigen
Nkx2.2: NK2 homeobox 2
Olig2: oligodendrocyte transcription factor 2
OPC(s): oligodendroglial precursor cell(s)
PBS: phosphate buffered saline
pcw: post-conceptional weeks
PDGF: platelet-derived growth factor
PDGFR: platelet-derived growth factor receptor
PFA: paraformaldehyde
PKD1: polycystic kidney disease 1/polycystin 1
PLD1: phospholipidase D1
Plp1: proteolipid protein-1
PRRG1: proline rich and gla domain 1
PRR5L: proline rich 5 like
RNA: ribonucleic acid
RNAseq: RNA sequencing
RPKM: reads per kilo base per million mapped reads
RT: room temperature
SLC44A1: solute carrier family 44 member 1
Sox10: sex-determining region Y-box 10
SVZ: subventricular zone
TAP: transiently amplifying progenitor
TBS(-T): Tris-buffered saline (supplemented with Triton)
TPM: transcripts per kilobase million
qRT-PCR: quantitative reverse transcription polymerase chain reaction
Wnt: wingless and integration site.
