# Supplementary material for: C21orf91 Regulates Oligodendroglial Precursor Cell Fate—A Switch in the Glial Lineage?
Source: Front Cell Neurosci. 2021 Mar 16;15:653075. doi: 10.3389/fncel.2021.653075 (PMC8008080; doi:10.3389/fncel.2021.653075)
Supplement: SUPPLEMENTARY FIGURE 1 — Multiple sequence alignment of human C21ORF91 and model organism orthologs performed with ClustalO and Jalview. [file Table_1.pdf]

Supplementary Material

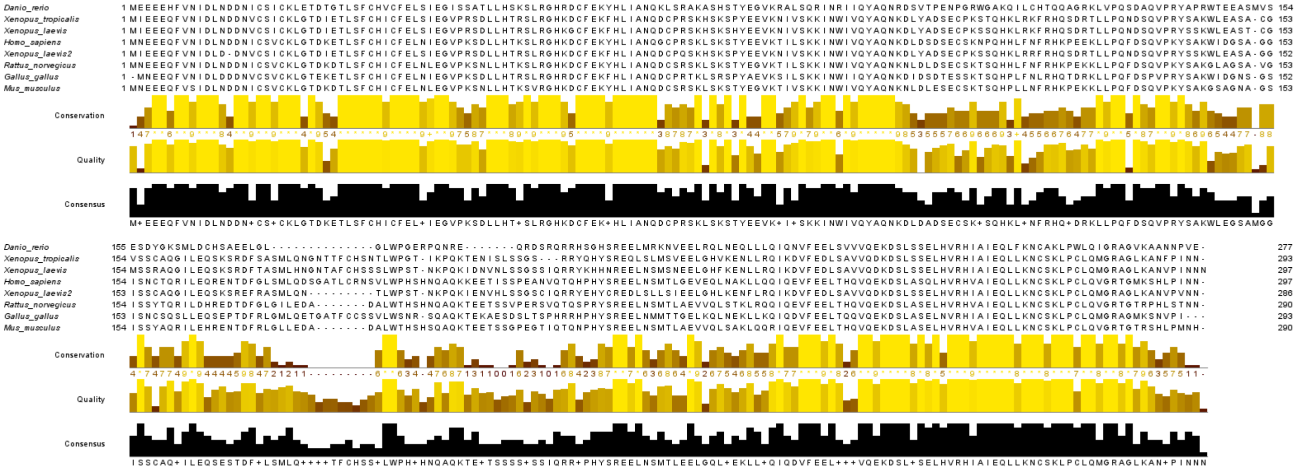

Supplementary Figure 1. Multiple sequence alignment of human *C21ORF91* and model organism orthologs performed with ClustalO and Jalview. *Danio rerio* Q503Y8, *Xenopus tropicalis* B3DL45, *Xenopus laevis* 2 A0A1L8HCS1, *Homo sapiens* Q9NYK6, *Xenopus laevis* A0A1L8H546, *Rattus norvegicus* D4ACQ8, *Gallus gallus* Q9I8W6, *Mus musculus* Q9D7G4.
